# Acute and Chronic Management of Neuromyelitis Optica Spectrum Disorder

**DOI:** 10.1007/s11940-015-0378-x

**Published:** 2015-10-03

**Authors:** Elena Sherman, May H. Han

**Affiliations:** Department of Neurology and Neurological Sciences, Stanford University, 1201, Welch Road, Stanford, CA 94305 USA

**Keywords:** Neuromyelitis optica, Treatment

## Abstract

Neuromyelitis optica and neuromyelitis optica spectrum disorder (NMO/NMOSD) is a rare but clinically aggressive demyelinating disease of the central nervous system (CNS) caused by antibodies against water channel protein aquaporin 4 (AQP4) in the astrocytic foot processes. Patients typically present with optic neuritis (ON) or longitudinally extensive transverse myelitis (LETM). The majority of patients with NMOSD show good response to treatment with steroids and plasmapheresis in the acute setting; however, 90 % of patients will eventually have clinical relapses and accrue permanent disability. Currently, immune modulation is the mainstay of maintenance therapy with anti CD-20 (rituximab, Rituxan™) having collectively the strongest evidence to support its use and mycophenolate mofetil having comparable reductions in absolute relapse rate (ARR) and expanded disability status scale (EDSS) scores. Azathioprine, mitoxantrone, and methotrexate also have retrospective case series data that demonstrate reduction in ARR and stabilization of EDSS but with higher relapse rates and exposure to greater risk of treatment toxicities. Excitingly, multiple novel therapies are under clinical study for patients who are refractory to these first-line therapies including monoclonal antibodies targeting interleukin-6 (IL-6), CD19, CD20, complement, and neutrophil elastase inhibitors which may provide additional options for patients with severe clinical presentations. Importantly, no randomized clinical trials have been published to date comparing clinical outcomes of different maintenance therapies in NMOSD. Several trials are currently underway, and results will help guide future management decisions as current evidence is from many small, retrospective case series and cohort studies with many potential confounds.

## Introduction

### Clinical presentation and epidemiology

Neuromyelitis optica spectrum disorder (NMOSD), formerly referred to as neuromyelitis optica (NMO) or Devic’s disease, is a demyelinating disorder thought to be caused by immunoglobulin G (IgG) antibodies targeting water channel protein aquaporin 4 (AQP4) located in astrocytic foot processes contributing to the formation of blood-brain barrier (BBB) [1]. Incidence of NMO is estimated at 0.05–4.4 per 100,000 with a 3:1–9:1 female predominance and typically presents in the third to fourth decade [2•]. The disease is more commonly seen among patients of Asian, African, and Hispanic ancestry; however, NMO occurs worldwide [3]. Clinical presentation most commonly consists of optic neuritis (ON), longitudinally extensive transverse myelitis (LETM) (>3 vertebral segments), or with an area postrema syndrome (APS). However, brain lesions are also seen in 60 % of patients, typically in periventricular locations along the third or fourth ventricles, in the thalamus, hypothalamus, or corpus callosum [4••].

It is of great importance to clinically differentiate NMO from more common demyelinating disorders such as multiple sclerosis (MS), as immunomodulatory therapies for MS including interferon-beta (IFN-β), glatiramer acetate, and natalizumab have been shown to be ineffective and, in some cases, harmful [4••]. Instead, immunosuppression with agents such as rituximab, mycophenolate mofetil (MM), and azathioprine (AZT) are considered first-line therapies as will be further described. Initiation of immune modulation is of paramount importance since 90 % of patients with NMOSD will relapse after initial presentation; 60 % will relapse in the first year [2•]. With each attack, patients acquire increasing levels of disability and often experience less recovery of function compared to patients with MS. Indeed, if untreated for 5 years, 50 % of patients will be wheelchair bound and functionally blind. With current treatments, at 5 years, 28 % require a cane to ambulate and less than 8 % are wheelchair bound [2•].

### Diagnostic criteria

Clinical diagnosis of NMO is based on international consensus diagnostic criteria that were published by Wingerchuk and colleagues earlier this year that can be met independent of AQP4 antibody positivity. If AQP4 IgG positive, a patient can meet criteria for NMO if at least one core clinical criteria is met (optic neuritis, acute myelitis, area postrema syndrome, acute brainstem syndrome, narcolepsy or diencephalic syndrome, or cerebral syndrome) with exclusion of alternative diagnoses to explain presentation. If AQP4 IgG negative, a diagnosis of NMO may be made with the presence of two of these core clinical criteria (one of which must be ON, LETM, or APS) with imaging appearance clinically consistent with NMO and without another alternative diagnosis to better explain presentation [4••]. These guidelines recommend that the term NMO be changed to NMOSD to be inclusive of patients with NMO features that may not be AQP4 IgG positive or may have presentations outside of the common ON and LETM presentations.

Despite these recently published recommendations, the 2006 Wingerchuk criteria continue to be used in ongoing research studies. According to these criteria, a definite diagnosis of NMO may be given if a patient presents with ON, acute myelitis, and at least two of three supportive criteria (transverse myelitis ≥3 vertebral segments, MRI brain not meeting diagnostic criteria for MS, and AQP4 IgG positivity [5]. Thus, NMOSD is a more inclusive term encompassing patients with features of NMO regardless of antibody status as well as those presenting with brainstem or brain lesions that are not encompassed by the 2006 criteria.

### Antibody markers

Anti-AQP4 antibodies are typically found in approximately 70 % of patients and screening assays are highly specific for the disorder, but with imperfect sensitivity estimated between 54 and 77 %, with higher sensitivity with newer cell-based assays [6]. AQP4 IgG seropositivity is associated with a more severe disease course and higher risk of relapses [3]. Antibodies to myelin oligodendrocyte glycoprotein (MOG) were present in 7.4 % of patients in a sample of 215 patients and were as a group younger, more likely to be male, to have a monophasic clinical presentation and to have better functional recovery [7]. Oligoclonal bands can also be helpful in differentiating NMOSD from MS as they are uncommon in NMO but can be seen in up to 20 % of patients [4••].

### Pathogenesis

As opposed to MS, the pathogenesis of NMO is mediated by the humoral immune system. Anti-AQP4 antibodies were identified in 2004 that bind to water channels located in astrocytic foot processes, the gray matter of the central spinal cord, and along periaqueductal and periventricular regions [1, 8, 9]. The distribution of AQP4 channels is correlated with common sites of disease activity in NMO, and experiments have demonstrated loss of AQP4 reactivity in active lesions which is thought to be caused by endocytosis of the AQP4 homotetramer after antibody binding [8]. AQP4 antibodies fix and activate complement which causes inflammation and tissue damage leading to disruption of the BBB, edema, and secondary demyelination [9]. In rats with experimental autoimmune encephalitis (EAE), administration of recombinant anti-AQP4 antibodies resulted in destruction of perivascular astrocytes and deposition of perivascular IgG and complement as is seen in humans, providing additional evidence for the role of AQP4 antibodies in the pathogenesis of NMO [10].

Understanding of this pathophysiology has led to treatments which target humoral immunity including monoclonal antibodies against B cells, rituximab, as well as more novel targets that are currently being investigated including antibodies against AQP4 antibody itself, neutrophil elastase, IL-6, complement, and CD59.

## Treatment

### Acute management

#### Corticosteroids

The mainstay of acute treatment for NMOSD is high-dose corticosteroids, typically solumedrol 1000 mg for 3–5 days based on standard therapy for other acute clinical presentations of demyelination. Corticosteroids mechanistically cause immunosuppression and anti-inflammation by decreasing peripheral lymphocytes, reducing inflammatory cytokines, and altering trafficking of leukocytes [11]. Solumedrol is generally well tolerated in a short-term setting, but it is important to provide ulcer prophylaxis and monitor for common psychiatric side effects including insomnia and agitation.

#### Plasmapheresis

Patients who do not clinically respond to an initial steroid pulse should be offered plasma exchange (PLEX) for five treatments, typically performed every other day. Some recent publications have advocated that PLEX be offered along with corticosteroids initially, especially in patients who present with relapse with prior response to PLEX [12]. Bonnan and colleagues retrospectively compared EDSS scores in 96 patients with NMO who received either steroids or steroids plus PLEX between 1982 and 2008 and found that the change in their EDSS at time of relapse compared 6 months after presentation was by 2.6 points in patients who received steroids plus PLEX versus 1.2 in patients who received steroids alone (*P* < 0.01) [13]. Other case series also report a clinical benefit from PLEX [14], and a recent comparison of steroids and steroids plus PLEX by Abboud and colleagues in 16 patients with NMO relapse showed 65 % of patients receiving steroids plus PLEX had a stable or improved EDSS post-relapse compared to only 35 % of patients who were treated with steroids alone [12].

### Maintenance therapies

Unlike MS, patients with NMO do not clinically respond to immunomodulatory therapies and may actually be harmed by their use [15, 16]. Instead, maintenance therapy is aggressively pursued through a number of immunosuppressant therapies, most commonly rituximab, MM, and AZT. At present, no randomized clinical trials have been performed to compare different immunosuppressive agents in terms of efficacy and current data is mostly limited to retrospective case series. Thus, clinical practice varies greatly in terms of region and practitioner experience.

Two recent studies allow for some limited comparisons of these agents. Mealy and colleagues performed a retrospective study comparing annual relapse rates (ARRs) in 90 patients receiving rituximab, MM, or AZT. Both MM and AZT patients received concomitant treatment with prednisone. On rituximab, median ARR decreased from 2.61 to 0.33 with 2/3 of patients achieving complete remission. On MM, median ARR decreased from 2.61 to 0.33 with 2/3 of patients also achieving complete remission. Overall, there was no statistically significant difference between ARR in patients treated with rituximab and MM, but both were superior to AZT [17••]. Another study by Torres and colleagues looked retrospectively at 71 patients with NMOSD to compare rituximab, MM, AZT, and cyclophosphamide with rituximab showing the greatest reduction in ARR (1.17 to 0.25, *P* < 0.01) but powered only to detect statistically significant differences in rituximab and AZT groups. Additionally, 50 % of patients receiving rituximab became remission free [18•].

**Fig. 1:**
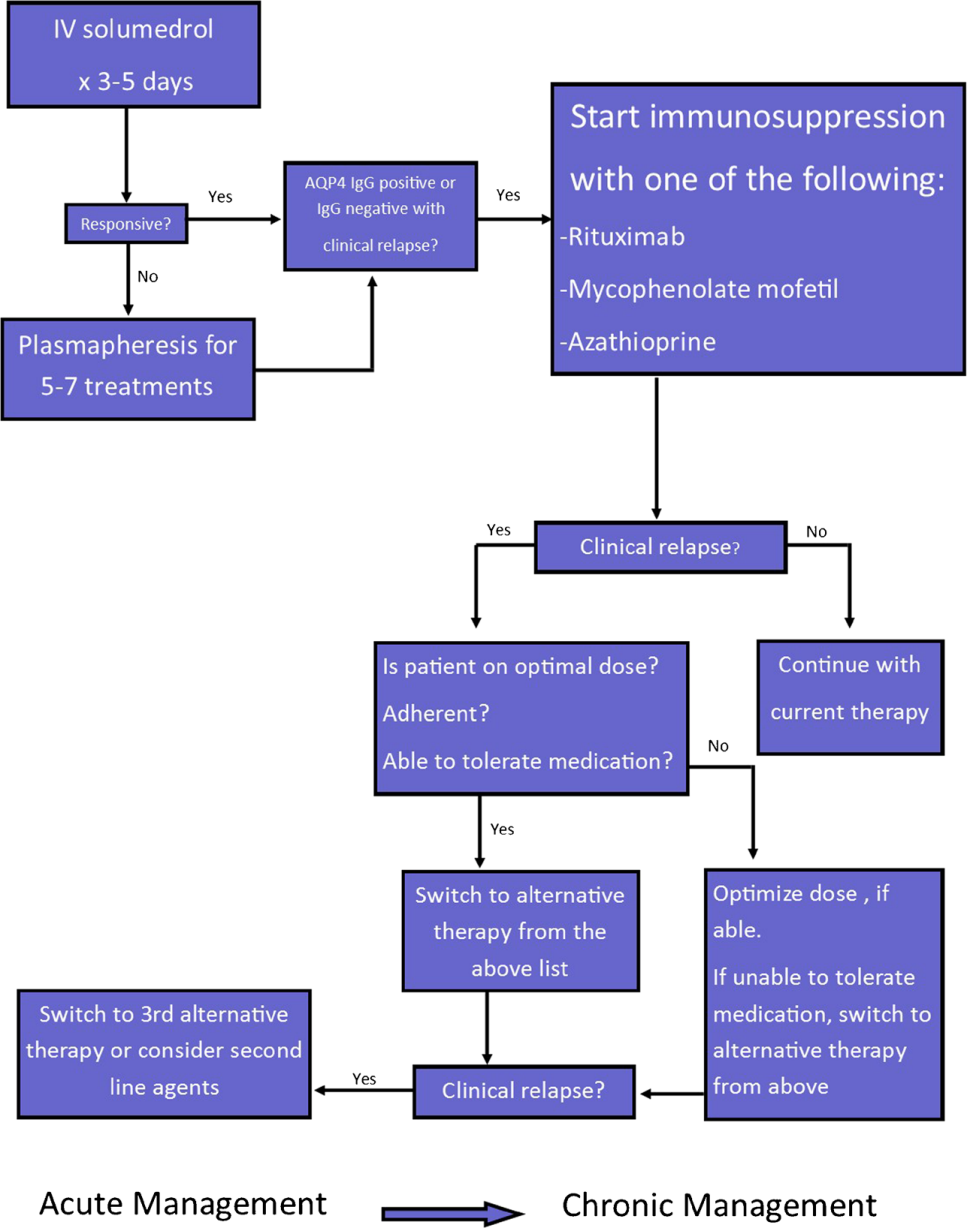
Treatment algorithm for acute and chronic management of NMOSD.

These studies are limited by retrospective, case series design, small sample size, and multiple confounders. Head-to-head randomized trials to compare these agents are needed in the future as agents vary greatly in terms of cost. Please refer to Table 1 for a summary of common medications used in chronic management of NMOSD including dosing and side effects. Figure 1 summarizes our recommended treatment algorithm based upon current literature.

**Table 1:** Common therapeutics in NMOSD

Agent	Initial dose	Maintenance dose	Mode of action	Side effects
Methylprednisolone	1000 mg daily for 3–5 days	N/A	Multiple	Insomnia, agitation, hypertension, hyperglycemia, ulcers
Plasmapheresis	5–7 cycles	N/A	Removal of AQP4 IgG and reduction of cytokines	Coagulopathy, hemodynamic instability
Rituximab	1000 mg weekly for 2 weeks or 375 mg/m^2^ weekly for 4 weeks	375 mg/m^2^ or 1000 mg weekly for 2 weeks when CD19 count >1 % on flow cytometry	Anti-CD20, B cell depletion	Sepsis, infections (Herpes zoster, UTIs, URIs), leukopenia, transaminase elevation, PML is rare
Mycophenolate mofetil	1000–2000 mg daily with concurrent prednisone (5–60 mg daily)	1000–2000 mg daily	Inhibits inosine monophosphate dehydrogenase, impairs B and T cell synthesis	Photosensitivity, recurrent infections, headache, constipation, abdominal pain, leukopenia, PML is rare
Azathioprine	2–3 mg/kg/day with concomitant prednisone (5–60 mg daily) for 6–12 months	2–3 mg/kg/day	Thiopurine antagonist of endogenous purines in DNA and RNA, interferes with lymphocyte proliferation	Nausea, diarrhea, rash, recurrent infections, leukopenia, transaminase elevation, increased risk of lymphoma
Mitoxantrone	12 mg/m^2^ for 3–6 months	6–12 mg/m^2^ every 3 months	Causes DNA cross-linking and strand breaks, interferes with DNA repair	Nausea, transaminase elevation, leukopenia, hair loss, amenorrhea, minor infections including UTI and URI, rarely heart failure and acute leukemia
Methotrexate	Start with 7.5 mg weekly with upward titration and concomitant prednisone (5–60 mg daily)	7.5–15 mg weekly with concurrent prednisone (5–10 mg daily for a least 6 months)	Folic acid antagonist	Pneumonitis, GI upset, cytopenia, hepatotoxicity
Cyclophosphamide	1000 mg every 2 months with associated steroids	Same	Cytotoxic alkylating agent, inhibits mitosis	GI symptoms, hyponatremia, heart block, pancytopenia, opportunistic infections

*CD* cluster of differentiation, *UTI* urinary tract infection, *URI* upper respiratory infection, *PML* progressive multifocal leukoencephalopathy.

#### Rituximab

Rituximab is a chimeric murine/human monoclonal antibody directed against CD20 antigen on B cells and precursor B cells. The first case series reporting benefit with use of rituximab in NMO patients was published by Cree in 2005 and noted a statistically significant reduction in the ARR from 2.6 to 0 in eight patients with 75 % of these patients remaining relapse free at 12 months. EDSS significantly decreased from 7 to 5.5 as a proxy for patient’s functional status [19]. This initial study was followed by multiple retrospective studies of various sizes demonstrating reduction in ARR and stabilization or improvement of EDSS with follow-up between 19 and 60 months [20, 21••, 22, 23].

Of note, each study used a different regimen in terms of induction and maintenance dosing as well as different parameters for timing of maintenance dosing. Regimens were based on either protocols for lymphoma or prior trials in MS. Interestingly, a small study in China of five patients reported that a dose of rituximab 100 mg for 3 weeks with repeat dosing when CD 19 cells >1 % (typically around 20 weeks) allowed all patients to remain remission free which begs the question of what an ideal therapeutic dose of rituximab should be, particularly given its high cost [22]. Kim et al. suggest that redosing should occur with rising of CD27+ memory B cells above 0.05 % in the first year of therapy and above 0.1 % thereafter as their peripheral depletion has been associated with clinical response to rituximab, but again larger studies examining these questions with randomized design are needed [21••].

**Standard dosage**: Induction with either 1000 mg weekly for 2 weeks or 375 mg/m^2^ weekly for 4 weeks followed by maintenance dose of either 375 mg/m^2^ or 1000 mg weekly for 2 weeks when CD19 count >1 % on flow cytometry
**Contraindications**: Severe liver disease, history of PML or prior chemotherapy
**Main side effects**: Sepsis, infections including Herpes zoster, UTIs, URIs, leukopenia, transaminase elevation, PML is rare
**Special points**: Cell counts should be monitored with flow cytometry every 3–6 months with additional maintenance dose given for CD19 count >1 %
**Cost/cost-effectiveness**: Expensive, approximately $27,000 per year

#### Mycophenolate mofetil

Mycopehnolate is a reversible inhibitor of inosine monophosphate dehydrogenase, an enzyme needed for synthesis of guanosine, and acts as an immunosuppressant by impairing B and T cell synthesis. As noted in the above studies, its reduction of ARR and EDSS are comparable to rituximab [17••]. A retrospective cohort of 24 patients was treated with 2000 mg daily with concomitant steroids resulted in a statistically significant reduction of ARR from 1.28 to 0.09 with stabilization or improvement in EDSS in 91 % of patients [24]. Twenty-five percent of patients experienced an adverse effect.

**Standard dosage**: 1000–2000 mg daily with concurrent prednisone (5–60 mg daily)
**Contraindications**: Allergy to medication
**Main side effects**: Photosensitivity, recurrent infections, headache, constipation, abdominal pain leukopenia, PML is rare
**Special points**: Goal absolute lymphocyte count < 1500, should monitor CBC every 1–3 months
**Cost/cost-effectiveness**: $2000 per year

#### Azathioprine

AZT is a thiopurine antagonist of endogenous purines in DNA and RNA and interferes with lymphocyte proliferation. AZT is one of the first-line immunosuppressant agents used for relapse prevention in NMOSD as well as many other autoimmune disorders. Multiple retrospective cohort studies have examined the efficacy of AZT in terms of ARR and EDSS with one study in 2015 showing a reduction of ARR from 0.92 to 0.56 in 22 patients with 32 % of subjects remaining relapse free [18•]. Another study in 2014 of 32 patients showed reduction of ARR by 72 %, but with over half of subjects continuing to have clinical relapses, which was inferior to outcomes on both rituximab and MM [17••]. Elsone and colleagues examined a sample of 103 patients treated with AZT and concomitant steroids with a statistically significant reduction of ARR from 1.5 to 0. Sixty percent of patients remained relapse free at 18 months, and EDSS stabilized or improved in 78 % [25]. A 2011 study by Costanzi and colleagues showed AZT +/− steroids led to a reduction in ARR from 2.19 to 0.64 over 22 months but with only 37 % of patients remaining relapse free [26].

Thus, AZT with initial concurrent steroids is a cost-effective alternative for immunosuppression in some patients but with higher risk of relapse.

**Standard dosage**: 2–3 mg/kg/day with concomitant prednisone (5–60 mg daily) for 6–12 months
**Contraindications**: Pregnancy, history of treatment with another alkylating agent, prior history of lymphoma
**Main side effects**: Nausea, diarrhea, rash, recurrent infections, leukopenia, transaminase elevation, increased risk of lymphoma
**Special points**: Typically start with a dose of 25 mg daily and titrate gradually to goal, can take 6 months to become biologically active so concomitant steroids needed during this period. Need to check thiopurine methyltransferase (TMPT) levels. If low, need to reduce dose by 50 % because of increased risk of myelosuppression. Monitor with monthly CBC.
**Cost/cost-effectiveness**: $2000 per year

#### Mitoxantrone

Mitoxantrone is an anthracenedione antineoplastic agent that works by intercalating with DNA, causing cross-linking and strand breaks and inhibits DNA repair by interference with topoisomerase I. Specifically, it inhibits migration of lymphocytes and decreases production of pro-inflammatory cytokines such as IL-2, TNF-alpha, and IFN-gamma which inhibit B cell function [27].

Two studies have examined its clinical efficacy in NMO for relapse prevention. The first was performed by Kim and colleagues and demonstrated a reduction of ARR from 2.8 to 0.7 in 20 patients with half of patients becoming relapse free [27]. No significant adverse effects were noted in this population. A second study in 2012 by Cabre and colleagues showed a statistically significant reduction in ARR from 1.82 to 0.37 over 5 years of follow-up with 32 % remaining relapse free at 5 years [28]. In this study, one patient developed mild CHF and one patient was diagnosed with AML leading to discontinuation of the drug in both cases.

**Standard dosage**: 12 mg/m^2^ for 3–6 smonths, then 6–12 mg/m^2^ every 3 months for maintenance
**Contraindications**: History of heart failure, leukemia, existing myelosuppression, hepatic impairment, pregnancy, renal disease
**Main side effects**: Nausea, transaminase elevation, leukopenia, hair loss, amenorrhea, minor infections including UTI and URI, rarely heart failure and acute leukemia
**Special points**: Should obtain baseline CBC, LFTs, uric acid level, and TEE prior to initiation of therapy. TEEs should be monitored prior to any maintenance dose to monitor LVEF and should continue to be monitored after discontinuation of therapy because of possibility of delayed cardiotoxicity.
**Cost/cost-effectiveness**: $4000 per year

#### Methotrexate

Methotrexate (MTX) is a folic acid antagonist that has been used as a maintenance therapy for NMO at some centers, although there is not a significant amount of data published on its use and outcomes. Kitley and colleagues published a retrospective observational case series of 14 patients that received MTX and steroids with reduction of ARR from 1.39 to 0.18 with stabilization of EDSS in 79 % [29]. Additionally, Ramanathan et al. observed a statistically significant reduction in ARR from 3.11 to 1.11 in 9 patients treated with MTX for 18 months with stabilization of EDSS in 5 patients [30].

**Standard dosage**: 7.5–15 mg per week with concomitant prednisone 5–60 mg daily for at least 6 months
**Contraindications**: Liver disease, pregnancy, severe renal impairment
**Main side effects**: Pneumonitis, GI upset, cytopenia, hepatotoxicity
**Special points**: Obtain baseline CXR, monitor LFTs, CBC, and renal function; concomitant folic acid therapy is required
**Cost/cost-effectiveness**: Relatively inexpensive compared to other immunosuppressants

#### Cyclophosphamide

Cyclophosphamide is a cytotoxic alkylating agent that has been used in NMO patients in two small clinical case series with contradictory findings. The first was of four Japanese patients with a reduction of EDSS from 8 to 5.75 after receiving 500 mg/m^2^ after IV steroids and PLEX after an acute attack [31]. A second study in 2012 showed no improvement in EDSS or ARR in seven patients [32].

**Standard dosage**: 1000 mg every 2 months with associated steroids
**Contraindications**: Pregnancy, liver disease, prior hypersensitivity reaction
**Main side effects**: Leukopenia, neutropenia, thrombocytopenia, arrhythmias, heart block, nausea/vomiting, hepatic sinusoidal obstruction syndrome, hyponatremia, pneumonitis, hematuria, opportunistic infections.
**Cost/cost-effectiveness**: $1400 per year

### Emerging therapies

In addition to the above immunosuppressant agents, novel therapeutics are currently undergoing investigation focusing upon multiple targets that are based upon our ongoing understanding of pathogenesis of NMOSD.

#### Tocilizumab

Tocilizumab is a human monoclonal antibody directed against the IL-6 receptor and has been previously used in rheumatoid arthritis and juvenile idiopathic arthritis. It is also currently used in NMOSD patients with aggressive presentations that have been refractory to rituximab or other immunosuppression [33]. Tocilizumab works by reducing plasmablast survival, thereby inhibiting AQP4 Ab production. Previous studies have shown increased IL-6 levels in serum and CSF of patients with NMO during relapses. The first published case of its use in NMO was in 2013 by Araki and colleagues with a single patient showing improvement in EDSS from 3.5 to 2 after four administrations [34]. This report was followed by a prospective pilot study of seven patients showing an improvement in ARR from 2.9 to 0.4 [35]. Another small retrospective case series of three patients saw similar improvement of ARR [36].

Most recently, a retrospective observational study of eight patients demonstrated improvement of ARR from 4.0 to 0.4 after receiving monthly infusions at doses of 6–8 mg/kg. All eight patients had experienced multiple relapses despite treatment with rituximab. Relapses did occur in five of the patients, but the majority occurred at a suboptimal dose or prolongation of the time interval between subsequent doses [33]. Adverse effects included elevated cholesterol levels, infections, venous thrombosis, and neutropenia.

**Standard dosage**: 8 mg/kg every 4 weeks
**Contraindications**: History of TB infection, liver disease, severe elevations in cholesterol, neutropenia, thrombocytopenia
**Main side effects**: GI disturbance, fatigue, UTIs, neutropenia, leukopenia, elevation of cholesterol, transient mild transaminase elevation, DVT, TB reactivation
**Special points**: Monitor lipids, CMP, and CBC every 6 weeks initially, then every 3 months after first 6 months of therapy. Need to initially screen for latent TB infection prior to initiation.
**Cost/cost-effectiveness**: $24,000 per year

#### Eculizumab

Eculizumab is a humanized monoclonal antibody directed against C5 protein which prevents its cleavage to C5a and C5b, the latter of which initiates the cytolytic terminal membrane attack complex (MAC) of the complement cascade. Complement deposition has been shown to be involved in the pathogenesis of NMOSD with product deposition co-localizing in areas where AQP4 is normally distributed [8].

To date, only one small, prospective clinical trial has been performed with eculizumab. Fourteen patients received 4 weeks of the drug initially and then were maintained with infusions every 2 weeks thereafter for 48 weeks. All remained relapse free at 1 year with the exception of two possible clinical relapses. ARR was reduced from 3.0 to 0 and median EDSS improved from 4.3 to 3.5 overall with all patients showing stabilization or improvement of their EDSS. Only one patient suffered an adverse effect of meningococcal sepsis and sterile meningitis [37]. A major prohibitive factor in using this treatment is its high cost relative to other therapies. A phase III open-label trial and randomized, double-blinded clinical trial are currently underway.

**Standard dosage**: IV 600 mg weekly for 4 weeks, then IV 900 mg every 2 weeks
**Contraindications**: Unresolved Neisseria infection
**Main side effects**: Headache, increased risk of infection with encapsulated organisms, especially meningococcal infections
**Special points**: Vaccinate with meningococcal vaccine at least 2 weeks prior to initiation
**Cost/cost-effectiveness**: $400,000 per patient-year, $7333 per 30 mg vial

#### Aquaporumab

Aquaporumab is a nonpathogenic recombinant human monoclonal antibody that is comprised of an Fc portion that tightly binds AQP4 and an Fc portion that is mutated to lack the potential to activate complement and cellular damage. In vivo and ex vivo experiments demonstrated that administration of aquaporumab prevented development of NMO lesions in a mouse model [38]. The drug is still undergoing preclinical development but theoretically would be an advantageous therapeutic target given lack of associated adverse side effects unlike other immunosuppressive agents.

#### CD19-targeted therapies

Rituximab has strong evidence to support its use in prevention of relapses in NMOSD, but up to 25–50 % patients continue to have clinical relapses despite adequate suppression of CD19 count [17••, 18•, 21••]. CD19 is expressed on plasmablasts responsible for AQP4 Ab production and their reconstitution after administration of rituximab was shown to be correlated with increasing serum AQP4 Ab levels [39]. Since an increase in CD19+ plasmablasts is correlated with relapse, CD19 represents an alternative B cell target with potential to be used alone or in conjunction with other monoclonal antibodies such as rituximab. Multiple CD19-targeted therapies are currently under development and being studied as treatments for leukemia (ALL and CLL), lymphomas (DLBCL, NHL), and autoimmune diseases including rheumatoid arthritis and lupus but could be investigated in NMOSD in the future [40].

#### Complement inhibitor CD59

CD59 is a glycophosphoinositol (GPI)-anchored membrane protein on astrocytes that inhibits that terminal C5b-C9 membrane attack complex [41]. There is current interest in studying upregulation of CD59 as a potential treatment for NMO because of previous studies in mice suggesting inhibition of CD59 led to worsening disease in NMO. In one study, murine astrocytes were exposed to both anti-AQP4 and anti-CD59 antibodies with increased complement-dependent cytotoxicity (CDC) to astrocytes in culture as well as ex vivo spinal cord slices [42]. The study also reported extensive longitudinal spinal demyelination in CD59 knockout mice thought to be related to increased CDC. CD59 pharmacological upregulation is not yet under clinical study in humans but represents a novel therapeutic approach warranting further investigation.

#### Granulocyte-targeted therapies

Pathological studies of NMO lesions are notable for IgM, IgG, and complement deposits that are co-localized in areas where AQP4 channels are typically found and tend to be perivascular. Antibody-dependent astrocyte damage including CDC leads to neutrophilic and eosinophilic inflammation [8]. Indeed, CSF of patients with NMO can be notable for a cellular pleocytosis, typically of <50 cells with neutrophilic and eosinophilic predominance [4••]. Several therapeutics are currently in development that target cellular inflammation in NMO including neutrophil elastase inhibitors and antihistamines.

Silvestat is a neutrophil elastase inhibitor that is involved in neutrophil migration and phagocytosis that is currently being used in Japan to treat acute respiratory distress syndrome [43]. Neutrophil elastase has been shown, along with other Th17 cytokines, to be elevated in patients with NMOSD [44]. The same authors used a mouse model of EAE induced by T helper 17 (Th17) cells with inflammation targeting the optic nerves and spinal cord as is seen in NMOSD to demonstrate that use of silvestat was correlated with reduced inflammatory infiltrates in both spinal cord and optic nerves [44]. Another study by Saadoun and colleagues observed reduced AQP4 loss and inflammation in neutropenic mice exposed to human AQP4 IgG and an improvement in lesion burden in mice exposed to intrathecal silvestat [45]. The authors postulate that silvestat could be helpful in treatment of acute NMO attacks as it could prevent migration of neutrophils into the CNS and thus prevent lesion formation.

Antihistamines, such as cetirizine, have also been found to reduce cytotoxicity mediated by AQP4 Ab and eosinophils in *in vivo* and *ex vivo* mouse models. In a mouse model, those mice exposed to intracerebral injection of AQP4 Ab and complement showed marked eosinophilic infiltration with lesions worsened in transgenic hypereosinophilic mice and decreased lesion burden in hypoeosinohilic mice (from gene deletion or exposure to IL-5) and in mice exposed to cetirizine [46]. Thus, antihistamines that can stabilize eosinophils may have future utility in treating NMOSD.

## Conclusions

The current management of NMOSD entails acute treatment with IV steroids as well as PLEX in patients without a significant response to steroids. Long-term management is important to prevent relapses and progression of disability with rituximab, MM, and AZT being the most common immunosuppressant agents currently in use. Current data investigating ARR and EDSS in patients taking immunosuppression is mostly limited to retrospective case series or cohort studies with limitations related to small study design, exposure to other concurrent therapies, and lack of randomization and control groups, but evidence is most supportive of rituximab and MM in small comparison studies. Randomized clinical trials including head-to-head comparisons of these medications are needed in the future to help determine optimal choice of therapy, particularly with varying cost of agents.

Excitingly, there are many emerging therapies currently under investigation including monoclonal antibodies directed toward elements of the complement cascade, IL-6, CD19+ plasma cells, and AQP4 itself, as well as therapeutics targeting granulocytes involved in inflammation in NMO and complement inhibitors. Development of a therapy with selective effect and reduced toxicity will be a central goal of future investigation.
